# Editorial: Dentistry and oral health in Africa

**DOI:** 10.3389/froh.2023.1160976

**Published:** 2023-03-03

**Authors:** Maha El Tantawi, Morenike Oluwatoyin Folayan, Olalekan Ayo-Yusuf

**Affiliations:** ^1^ Faculty of Dentistry, Alexandria University, Alexandria, Egypt; ^2^ Department of Child Dental Health, Obafemi Awolowo University, Ife, Nigeria; ^3^ School of Health Systems and Public Health, University of Pretoria, Pretoria, South Africa

**Keywords:** Africa, oral health, dentistry, north–south, south–south

**Editorial on the Research Topic** Dentistry and oral health in Africa

One of the challenges facing oral health is the scarcity of data about the distribution of oral diseases, risk indicators, workforce training and healthcare services (1). In the absence of oral health surveillance systems in most African countries, oral health researchers and academic institutions have additional responsibility to provide the much-needed evidence to guide decision making and policy development. Data is also needed because available evidence is often from other countries and does not address contextual factors that drive the risk for poor oral health in Africa. This Research Topic aims to contribute to the slowly growing body of literature on oral health problems in countries in Africa with focus on driving publications where there are limited data on oral health.

Contributors to this Research Topic provided information on diverse subjects of interest such as high-technology, oral health research fields requiring investments in infrastructure and equipment that may not be available in many institutions with limited resources in Africa. Sudi et al. gave an overview of the challenges facing researchers in Africa in oral genomics and emphasized on the importance of collaboration with international researchers to overcome some of these challenges. Genomics may help prevent oral disease with growing burden in Africa due to the high rate of population growth. The oral microbiome is another field that needs advanced equipment and materials. Kalanzi et al. characterized the difference in oral microbiota by the severity of dental caries using the DMFT index in HIV positive people and compared this with individuals negative for HIV using 16S rRNA gene sequencing. Although Africa is home to countries with high burden of HIV (2), there is limited data on the oral microbiome in people living with HIV in these countries.

Among the challenges facing dental practice in African countries is the need to ensure oral health within the social and healthcare system contextual background in Africa. Payment for dental care in most African countries is out of pocket with minimal availability of dental insurance (1). This may limit the possibility to provide periodic care, especially for relatively costly procedures such as prosthodontic appliances. Adam and Kimmie-Dhansay highlighted the high prevalence of denture stomatitis resulting from years of denture use by edentulous people in South Africa. The relatively high cost of new dentures may be a risk factor for long years of using the same set of dentures with high risk for stomatitis. Although South Africa is an upper-middle income country, it has one of the greatest levels of income inequalities worldwide. Universal health coverage (UHC) may address the impact of cost on the utilization of healthcare services and eventually, on oral health (1) in South Africa and many other African countries. The findings of this study emphasize the importance of accelerating the implementation of UHC and the inclusion of oral healthcare in it to support care provision in many African countries (3). Another challenge facing dental practice is the large numbers of refugees and internally displaced people. Chandler and her team identified the growing need for forensic dentistry to help manage the problem of undocumented migrants resulting from humanitarian crises in many countries in Africa. Forensic dentistry helps identify people's identity and teeth record information that remains even in death (4). Currently, there is limited training on forensic dentistry in dental schools in Africa. However, this area of practice may assume increasing importance because of the growing humanitarian crises in Africa due to poverty, armed conflict, climate change, political instability, and food insecurity (5). For example, the Sahel region of Africa has one of the fastest growing humanitarian crises globally with an increase of more than 25% of people in need of life-saving assistance between 2017 and 2022 (5). Also, it is estimated that about 21.8 million people are internally displaced in African countries (6) with impact on oral health (Joury) (7). Yet, the priority areas of the Science, Technology and Innovation Strategy for Africa 2063 currently has no reference to oral health (8). A collective African strategy is needed to improve oral health in the region.

Several recommendations can be based on the findings in this Research Topic. First, it is important to establish opportunities for multi-department/ multidisciplinary collaborative research between universities and researchers in Africa. All the studies in this Research Topic were outputs from multiple authors which help address oral problems. Second, North-South partnerships help address oral health problems in resources-limited settings in Africa using state of the art technology and know-how from institutions in resources-rich settings. In addition, South-South collaboration resulting in multi-African countries authorship is also needed. Oral health researchers on the continent need to collaborate to drive the generation of evidence to inform the development of policies, guidelines and programs that will reduce the growing burden of oral diseases in Africa. Third, the growing humanitarian crisis on the continent needs attention, not only for the dead through the training of forensic dentists, but also for the living by investing in manpower and infrastructural development to improve the quality of life of people living in Africa.
